# Huaiqihuang (HQH) granule alleviates cyclophosphamide-induced nephrotoxicity via suppressing the MAPK/NF-κB pathway and NLRP3 inflammasome activation

**DOI:** 10.1080/13880209.2021.1990356

**Published:** 2021-10-25

**Authors:** Yueming Zhang, Jian Chang, Huan Gao, Xiaoyu Qu, Jinghui Zhai, Lina Tao, Jingmeng Sun, Yanqing Song

**Affiliations:** a Department of Pharmacy, the First Hospital of Jilin University, Changchun, China; b Department of Pediatric Oncology, the First Hospital of Jilin University, Changchun, China

**Keywords:** Chinese medicine, chemotherapeutic agent, oxidative stress, apoptosis, inflammation

## Abstract

**Context:**

Severe nephrotoxicity greatly limits the clinical use of the common effective chemotherapeutic agent cyclophosphamide (CYP). Huaiqihuang (HQH) is a Chinese herbal complex with various pharmacological activities, widely used for treating kidney disease.

**Objective:**

This study estimates the protective effect of HQH against CYP-induced nephrotoxicity in rats.

**Materials and methods:**

Four groups of 10 Sprague-Dawley rats were pre-treated with once-daily oral gavage of 3 and 6 mg/kg HQH for 5 days before receiving a single dose of CYP (200 mg/kg i.p.) on the 5th day; the control group received equivalent dose of saline. Renal function indices, morphological changes, oxidative stress, apoptosis and inflammatory mediators were measured. In addition, phosphorylation of the NF-κB/MAPK pathway and the activation of the NLRP3 inflammasome were analysed.

**Results:**

Both doses of HQH reduced the levels of serum creatinine (31.27%, 43.61%), urea nitrogen (22.66%, 32.27%) and urine protein (12.87%, 15.98%) in the CYP-treated rats, and improved histopathological aberrations. Additionally, HQH decreased the production of MDA (37.02%, 46.18%) and increased the activities of antioxidant enzyme CAT (59.18%, 112.25%) and SOD (67.10%, 308.34%) after CYP treatment. HQH protected against CYP-induced nephrotoxicity by modulating apoptosis-related protein and suppressing the inflammatory responses. Furthermore, the phosphorylation of the NF-κB/MAPK pathway and the activation of the NLRP3 inflammasome were significantly boosted in CYP-treated rats, which was also abrogated by HQH treatment.

**Conclusions:**

HQH effectively protected against CYP-induced nephrotoxicity, which was associated with regulating oxidative stress, apoptosis and inflammation, and so HQH may be a useful agent for treating nephrotoxicity caused by CYP.

## Introduction

Cyclophosphamide (CYP) is an alkylating anticancer drug and a potent immunosuppressant, extensively used in therapies of malignancies such as solid tumours and lymphomas, and nonneoplastic diseases, such as rheumatoid arthritis and systemic lupus erythematosus since the 1950s (Ahlmann and Hempel 2016; Jiang et al. 2019). Unfortunately, accompanied by beneficial effects, the application of CYP often causes severe nephrotoxicity, which is the major limiting factor in its use (El-Shabrawy et al. 2020; Jiang et al. 2020; Waz et al. 2021). The specific mechanisms of CYP-induced renal damage have been found to associate with oxidative stress, apoptosis and inflammation (Nagi et al. 2011; Mohammadi et al. 2014; Sherif 2018). Excessive oxidative awakened the phosphorylation of nuclear factor kappa-B (NF-κB) and its upstream mitogen-activated protein kinase (MAPK) pathways (Lawrence 2009; Kim and Choi 2010). Additionally, the nucleotide-binding domain (NOD)-like receptor protein 3 (NLRP3) inflammasome, comprises the NLRP3 protein, caspase-1 and apoptosis-associated speck-like protein containing a CARD (ASC), plays a vital role in the development of various inflammatory diseases including kidney injury, and it is a potential therapeutic target (Kim et al. 2018, 2019). Thus, the approaches that regulate these pathophysiological processes may have potential to reduce nephrotoxicity derived from CYP. However, there are still no effective strategies for relieving these detrimental effects. Therefore, novel treatments need to be identified.

Huaiqihuang (HQH), a classic Chinese herbal complex, mainly composed of *Trametes robiniophila* Murr. (Auriculariacaee)*, Lycium barbarum* Linn (Solanaceae) and *Polygonatum sibiricum* Delar. ex Redoute (Liliaceae). These components have been used extensively in China for thousands of years and proven to be effective in alleviating renal injury (Geng et al. 2015; Pan et al. 2019). HQH possesses significant antioxidant, anti-apoptosis and anti-inflammatory potential and exhibits a therapeutic effect against kidney diseases. A randomized controlled trial reported that HQH effectively reduced proteinuria and haematuria in patients with mild IgA nephropathy (Li et al. 2013). Basic experiments demonstrated a direct protective link between HQH and intrinsic renal cells. An *in vitro* study showed that HQH could protect renal tubular epithelial cells by slowing down the epithelial-mesenchymal transition (Pu et al. 2016). In addition, HQH exhibited beneficial effects against hyperglycemia-induced MPC5 podocyte dysfunction by inhibiting mitochondrial dysfunction, endoplasmic reticulum stress, and the apoptotic signalling pathway (Li et al. 2017). Importantly, HQH can reduce the toxicity of the drug after treatment. Zhu et al. (2011) reported HQH can significantly reduce doxorubicin-induced urinary excretion of proteins, decreased tubulointerstitial damage, and protected glomerular podocytes in rats by inhibiting inflammatory cytokine expression and macrophage infiltration. HQH also has a beneficial effect similar to glucocorticoid on cisplatin-induced primary nephritic syndrome (Guo et al. 2018). It is noteworthy that HQH exhibits almost no toxicity towards normal kidney cells, unlike many other drugs, and it can be long-term used. Therefore, HQH is very likely to be an auxiliary drug for treating kidney disease in the clinic.

Although HQH has achieved satisfactory results against renal disease, its effects in CYP-induced nephrotoxicity remains unclear. This study aimed to investigate the protective effects of HQH in renal injury caused by CYP and the possible molecular mechanisms involved. The findings may provide a theoretical basis for the use of HQH in treatment of CYP-induced nephrotoxicity in the clinic.

## Materials and methods

### Animals

**Figure 1.**
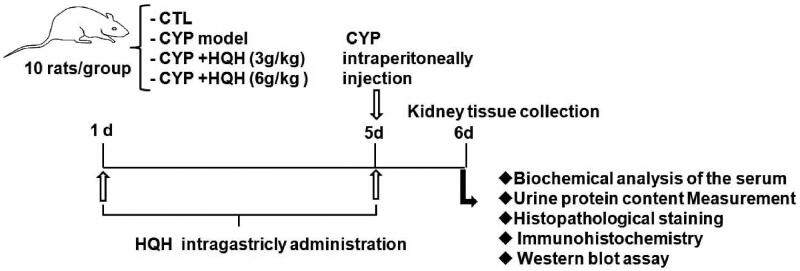
Rat treatment and experimental flow chart.

Male Sprague-Dawley rats (200 ± 20 g, 6 weeks old) were purchased from the Experimental Animal Centre of Jilin University (Jilin, China). All animal handling procedures were based on the National Institutes of Health Guide (NIH Publication No. 85-23, revised 2011) and approved by the Animal Ethics Committee of Jilin University. The animals were placed in a standard animal room with constant temperature (22 ± 3 °C) and humidity (50 ± 10%), a 12 h light/dark circle, and free access to food and water. After housing 7 days, the rats were randomly divided into the following 4 groups (10 per group): control group, CYP-only group, CYP + low-dose HQH (3 g/kg) group, CYP + high-dose HQH (6 g/kg) group. The control group received equivalent dose of saline; HQH (3 or 6 g/kg) was administered intragastrically once daily for 5 consecutive days. On day 5, CYP was injected intraperitoneally at a single dose of 200 mg/kg at 1 h before the final HQH treatment. At 24 h after the final treatment, the rats were anaesthetized and sacrificed. Blood samples and kidney tissues were collected for further experimental analysis (Figure 1).

### Assessment of serum enzymes

The blood samples collected in tubes were centrifuged at 3000 *g* for 10 min to obtain the serum. The serum level of creatinine (CRE), blood urea nitrogen (BUN), malondialdehyde (MDA), superoxide dismutase (SOD) activity, and catalase (CAT) activity were then determined spectrophotometrically according to the instructions of the assay kits (Nanjing Jiancheng Bioengineering Institute, Nanjing, China).

### Histological examination and immunohistochemistry

The kidney tissues were fixed in 4% formalin for 24 h and dehydrated using a graded series of alcohol (70%, 90%, and 100%), embedded in molten paraffin wax, cut into 5 mm slices, stained with haematoxylin and eosin (H&E) and assessed by a pathologist who was blinded to animal treatments. The kidney injury score was analysed on a five-point scale: 0 = no damage, 1 = 10% of the histopathology damage, 2 = 10–25% damage, 3 = 25–50% damage, 4 = 50–75% damage, 5 = more than 75%. For immunohistochemical analysis, the sections were incubated with primary anti-NLRP3 first antibody and secondary anti-rabbit IgG antibody in combination with 3,3′-diaminobenzidine (DAB) followed by haematoxylin counterstain. All preparations were observed at 100× and 400× magnification with a light microscope (Nikon Eclipse TE2000-U, Nikon, Japan) to detect the changes.

### Assessment of urine protein content

Rat urine samples were collected in tubes and protein content was determined spectrophotometrically according to the instructions of the kits (Nanjing Jiancheng Bioengineering Institute (Nanjing, China).

### Western blotting analysis

The tissues were solubilized using RIPA lysis buffer containing 1% phenylmethanesulfonyl fluoride for protein extraction. Total protein lysates received 15 min centrifuge at 4 °C with 13500 *g* and the protein concentration of the collected supernatant was assayed by bicinchoninic acid (BCA). Western blotting was conducted according to previous reports. In brief, proteins were separated by 8% or 12% precast gels and electrophoretically transferring to polyvinylidene fluoride membrane. The membranes were first blocked in TBST buffer (Tris-Hcl, NaCl, Tween20) with 5% skim milk powder at room temperature for 1 h and incubated with primary antibody against phospho p38 MAPK, total p38 MAPK, phospho JNK, total JNK, NLRP3, ASC, caspase1, Bcl-2, Bax, caspase-3, caspase-9, NFκB/p65, TNF-a, IL-6, IL-1β and GAP (1:1000) at 4 °C overnight. The membranes were then washed and incubated with secondary antibody (1:1000) at room temperature for 1 h. After washing three times with TBST, the target proteins were detected using enhanced chemiluminescence reagent (Yeasen Biotech, Shanghai, China) and quantitated by Image J software.

### Statistics

The data were expressed as mean ± standard deviation (SD). The differences between groups analysis were assessed with one-way ANOVA followed by Dunnett’s test or Student’s *t*-test. *p* value < 0.05 indicates the statistically significant difference. All the experiments were repeated at least three times.

## Results

### HQH alleviated renal damage in CYP-treated rats

**Figure 2.**
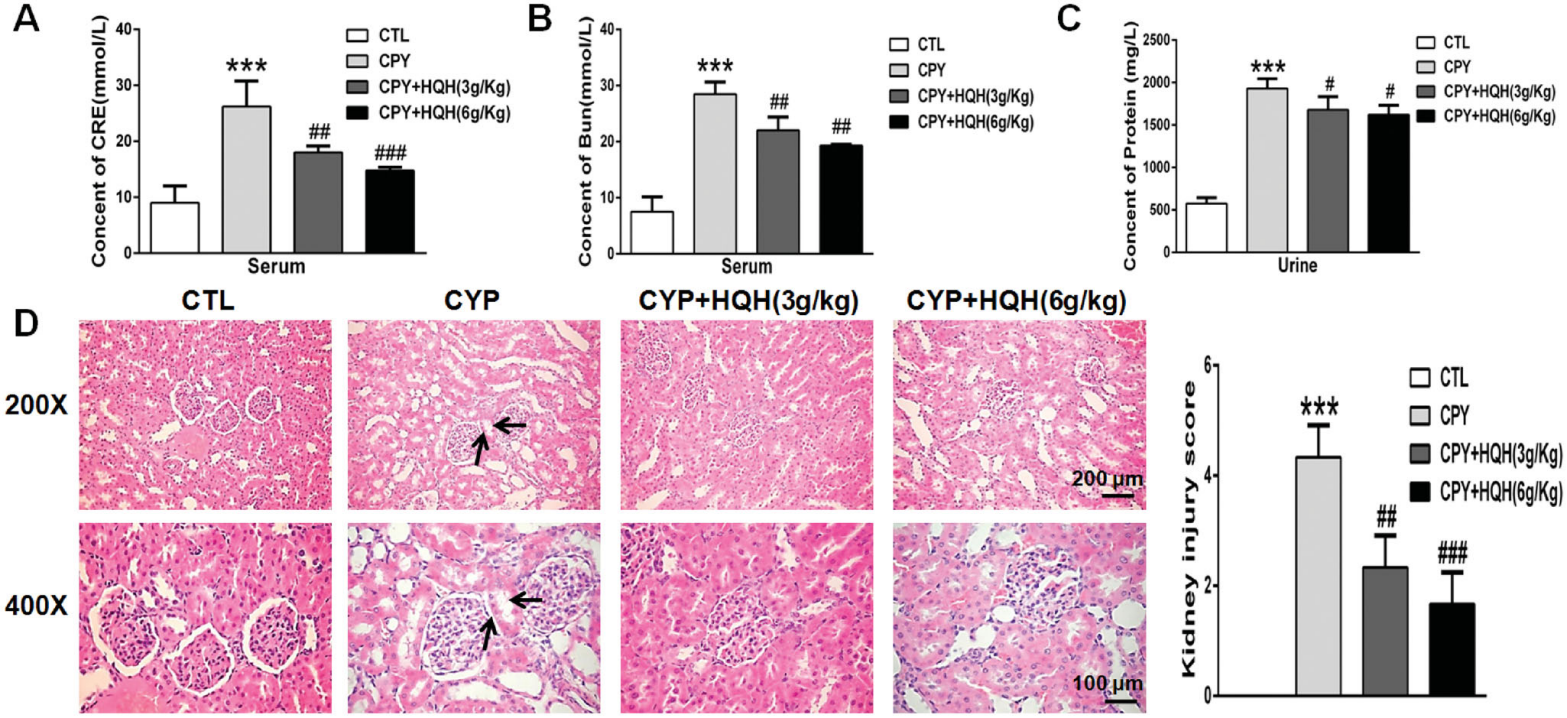
Effects of HQH on functional marker and morphological change in the kidneys of CYP-treated rats. (A) Serum creatinine. (B) Blood urea nitrogen. (C) Urine protein. (D) Kidney sections stained with H&E (magnification ×200 and ×400). The data are presented as the mean ± SD. *n* = 4. ***p < 0.001 versus the Control group. #*p* < 0.05, ##*p* < 0.01, and ###*p* < 0.001 versus the CYP group.

To investigate the protective effects of HQH against CYP-induced rat kidney injury, the levels of serum BUN and CRE and urine protein were tested to measure the renal function, meanwhile, H&E staining assay were performed to detect the changes of histopathology in kidney. As shown in Figure 2(A–C), compared to the levels of control group, CYP obviously increased the levels of BUN, CRE and urine protein by 3.79-, 2.90- and 3.35-fold, respectively, and the pre-treatment of HQH fully suppressed these abnormal changes. The effect of HQH treatment on renal histopathology is presented in Figure 2(D), the result showed that kidney architecture was normal in the control group, whereas the CYP group showed obvious pathological alterations including protein tubules, nuclear shrinkage and tubular vacuolisation, which were evidently restored by pre-treatment with HQH. Taken together, these results revealed that HQH relieved CYP-induced kidney damage in rats.

### HQH mitigated CYP-induced renal oxidative stress

**Figure 3.**
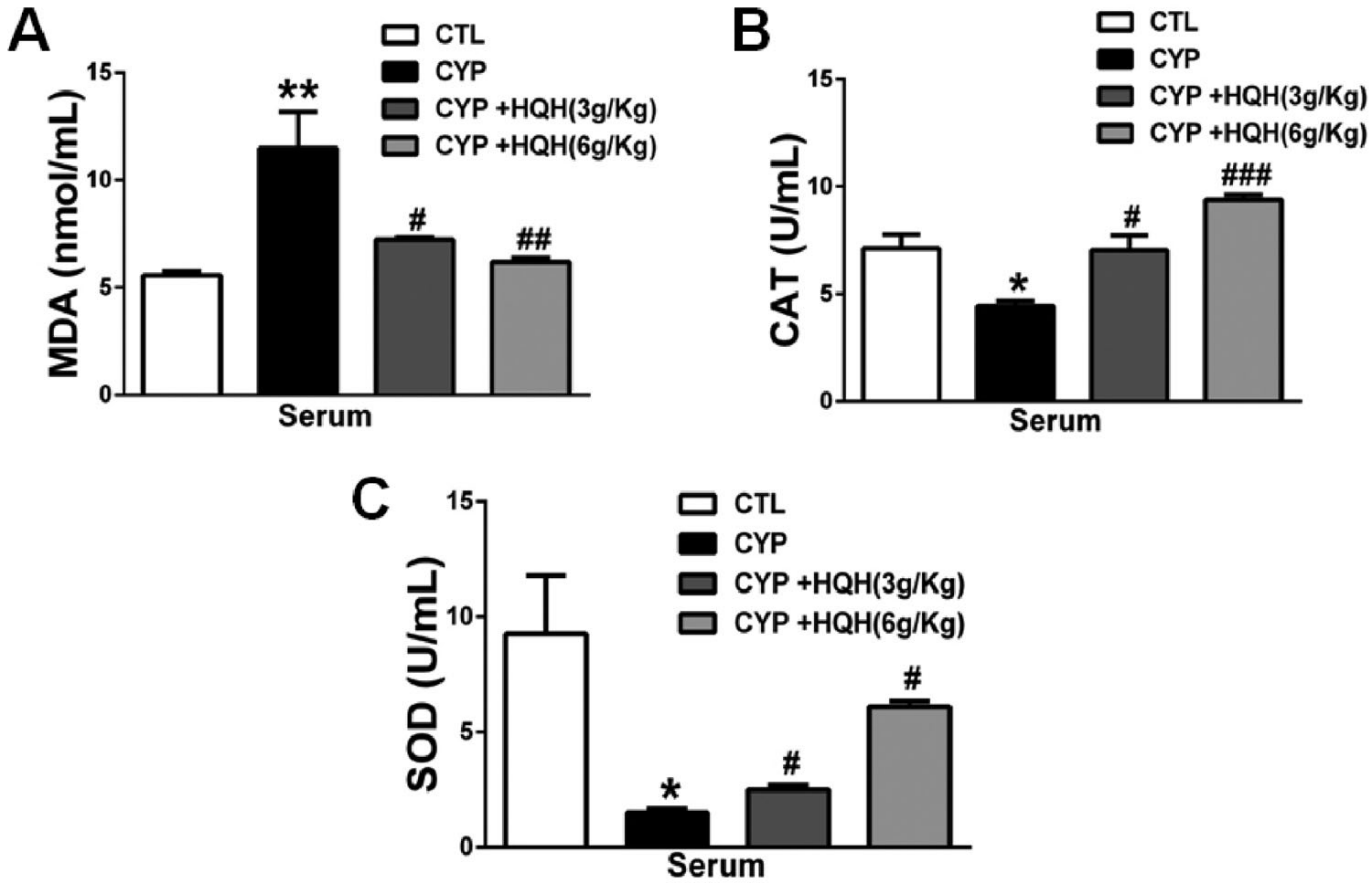
Effect of HQH on MDA levels and antioxidant enzyme activity in serum. (A) MDA levels. (B) CAT activity. (C) SOD activity. Data are shown as mean ± SD, *n* = 4. *p < 0.05 and **p < 0.01 versus the Control group. #*p* < 0.05, ##*p* < 0.01, and ###*p* < 0.001 versus the CYP group.

To explore the effect of HQH on oxidative stress, the level of the endogenous lipid peroxidation marker MDA and the antioxidant activities of SOD and CAT in serum of CYP-treated rats were detected. As shown in Figure 3, in comparation with control group, MDA content was significantly increased by 2.06-fold and the activities of SOD and CAT were significantly reduced by 84% and 38% in CYP group. However, coadministration of HQH with CYP ameliorated these negative changes compared to the CYP-only group.

### HQH reduced kidneys apoptosis in CYP-treated rats

**Figure 4.**
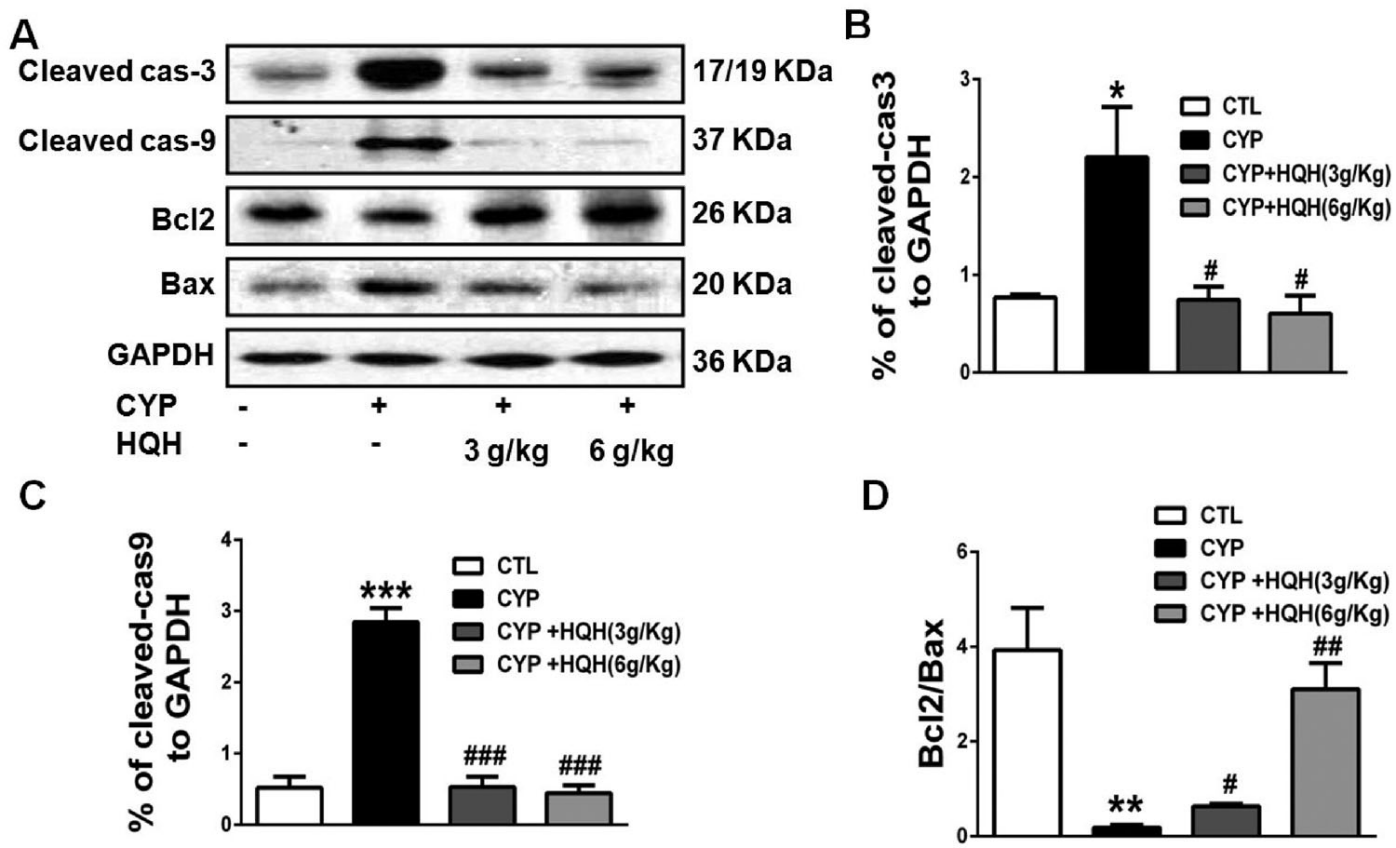
Effect of HQH on the apoptosis-related protein levels in the kidneys of CYP-treated rats. Western blot was used to test the protein expressions. (A) Representative images of cleaved-caspase3, cleaved-caspase9, Bcl2 and Bax were shown. (B) The levels of cleaved-caspase3. (C) The levels of cleaved-caspase9. (D) The ratio of Bcl2/Bax. Data are shown as mean ± SD, *n* = 4. *p < 0.05 and ***p < 0.001 versus the Control group. #*p* < 0.05, ##*p* < 0.01 and ###*p* < 0.001 versus the CYP group.

Research has revealed that CYP treatment promoted the level of apoptotic protein in mice (Jiang et al. 2020). To evaluate the anti-apoptotic role of HQH in CYP-treated rats, we determined the expression of caspase-3, caspase-9, and the ratio of the pro-apoptotic protein Bax to the anti-apoptotic protein Bcl-2 by western blot ting analysis (Figure 4). The result showed that CYP treatment significantly increased the Bax/Bcl-2 ratio, the expression of apoptosis effector caspase-3 and apoptosis initiator caspase-9 by 21.53-, 2.87- and 5.47-fold in kidney tissues, and these changes were clearly reversed by HQH.

### HQH abated kidney inflammation in CYP-treated rats

**Figure 5.**
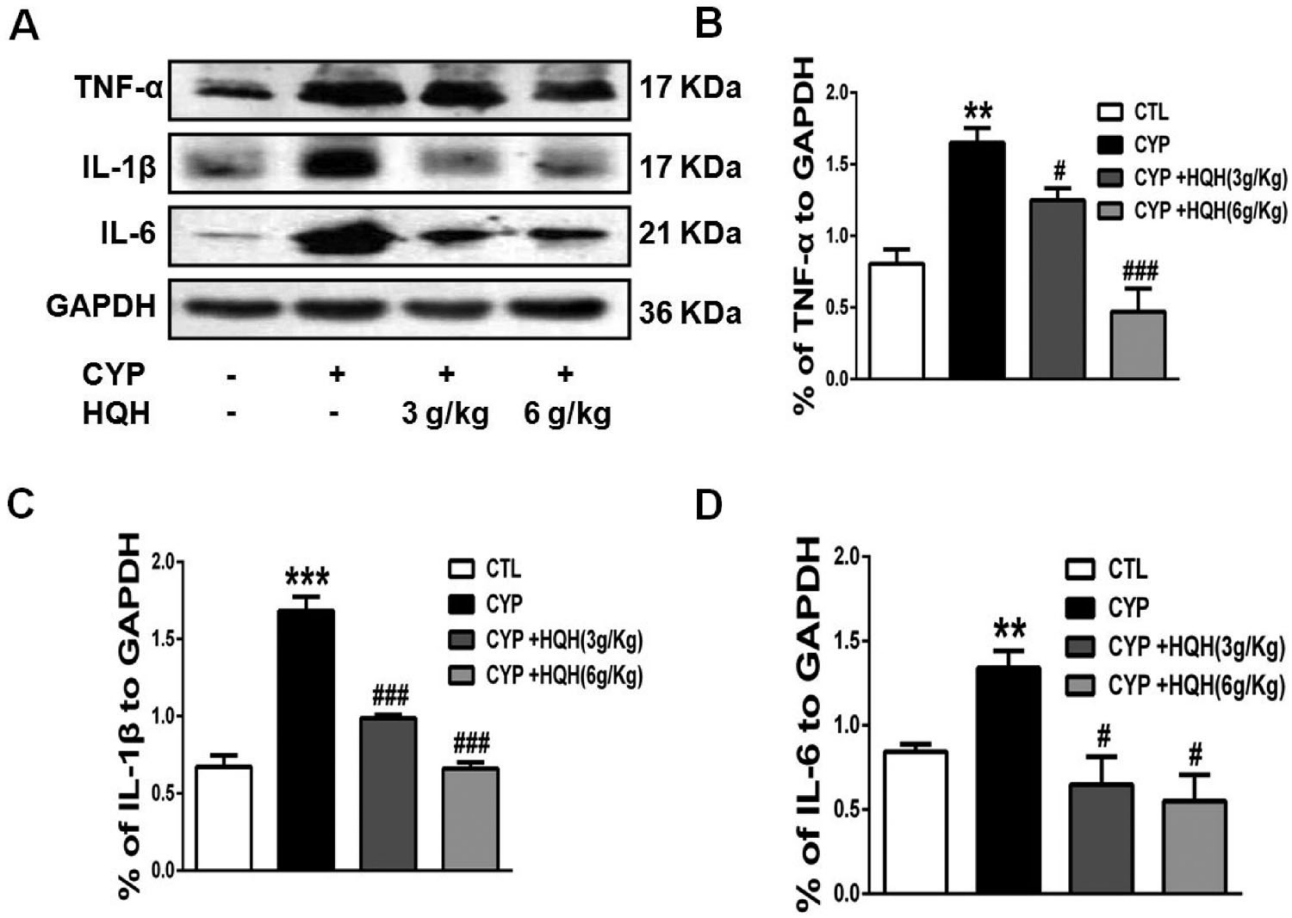
Effect of HQH on the inflammatory cytokines levels in the kidneys of CYP-treated rats. The expressions of TNF-α, IL-1β and IL-6 were determined by western blot (A) The levels of TNF-α. (B) The levels of IL-1β. (C) The levels of IL-6. Data are shown as mean ± SD, *n* = 4. **p < 0.01 and ***p < 0.001 versus the Control group. #*p* < 0.05 and ###*p* < 0.001 versus the CYP group.

The effect of HQH on kidney inflammation was determined by western blotting. The pro-inflammatory cytokine levels (IL-1β, IL-6, and TNF-α) was increased by 2.50-, 1.59- and 2.05-fold in renal tissues in rats following CYP induction showed a significant increase in renal tissues when compared to the levels in control rats (Figure 5). However, these negative increases caused by CYP were significantly attenuated by HQH pre-treatment.

### HQH suppressed the phosphorylation of MAPK/NF-κB signalling pathways in kidneys in CYP-treated rats

**Figure 6.**
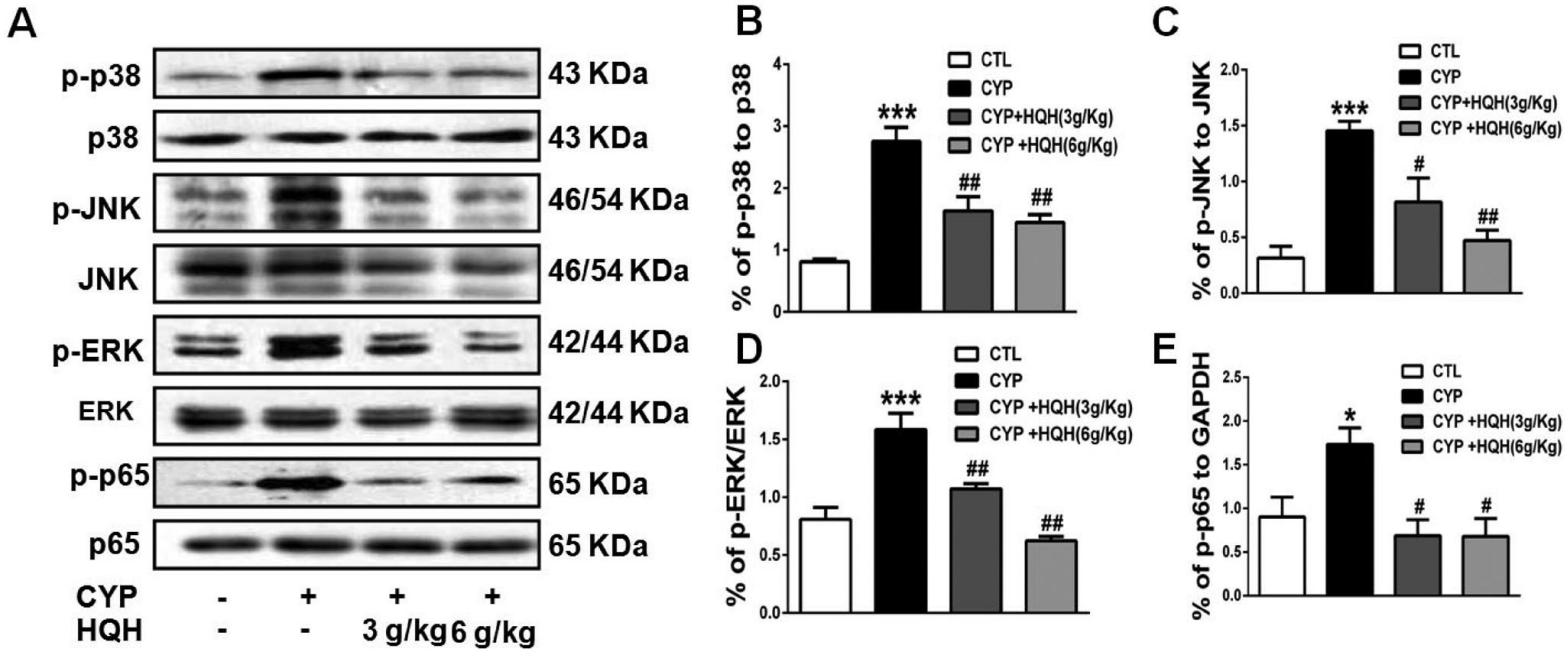
Effect of HQH on protein expression in the MAPK/NF-κB pathway in the kidneys of CYP-treated rats. (A) Phosphorylated p38, JNK, ERK and p65 expression was determined by western blot and representative images of protein bands were shown. (B) The levels of p-p38. (C) The levels of p-JNK. (D) The levels of p-ERK. (E) The levels of p-p65. Data are shown as mean ± SD, *n* = 4. *p < 0.05 and ***p < 0.001 versus theControl group. #*p* < 0.05 and ##*p* < 0.01 versus the CYP group.

Activation of MAPK/NF-κB signalling has long been thought to play a pivotal role in the inflammatory response by modulating the expression of pro-inflammatory cytokines. Hence, we investigated whether MAPK/NF-κB signalling was involved in the protective effect of HQH against CYP-induced kidney injury. The protein levels of phosphorylated and total JNK, ERK, p-38 and NF-κB 1.92 were tested by western blotting and the results unveiled that the phosphorylation of JNK, ERK, p-38 and NF-κB proteins increased by 4.63-, 1.98-, 3.39- and 1.92-fold in the kidneys of rats treated with CYP compared with control group. HQH treatment significantly decreased JNK, ERK, p-38 and NF-κB phosphorylation (Figure 6(A–D)).

### HQH inhibited NLRP3 signalling in kidneys in CYP-treated rats

**Figure 7.**
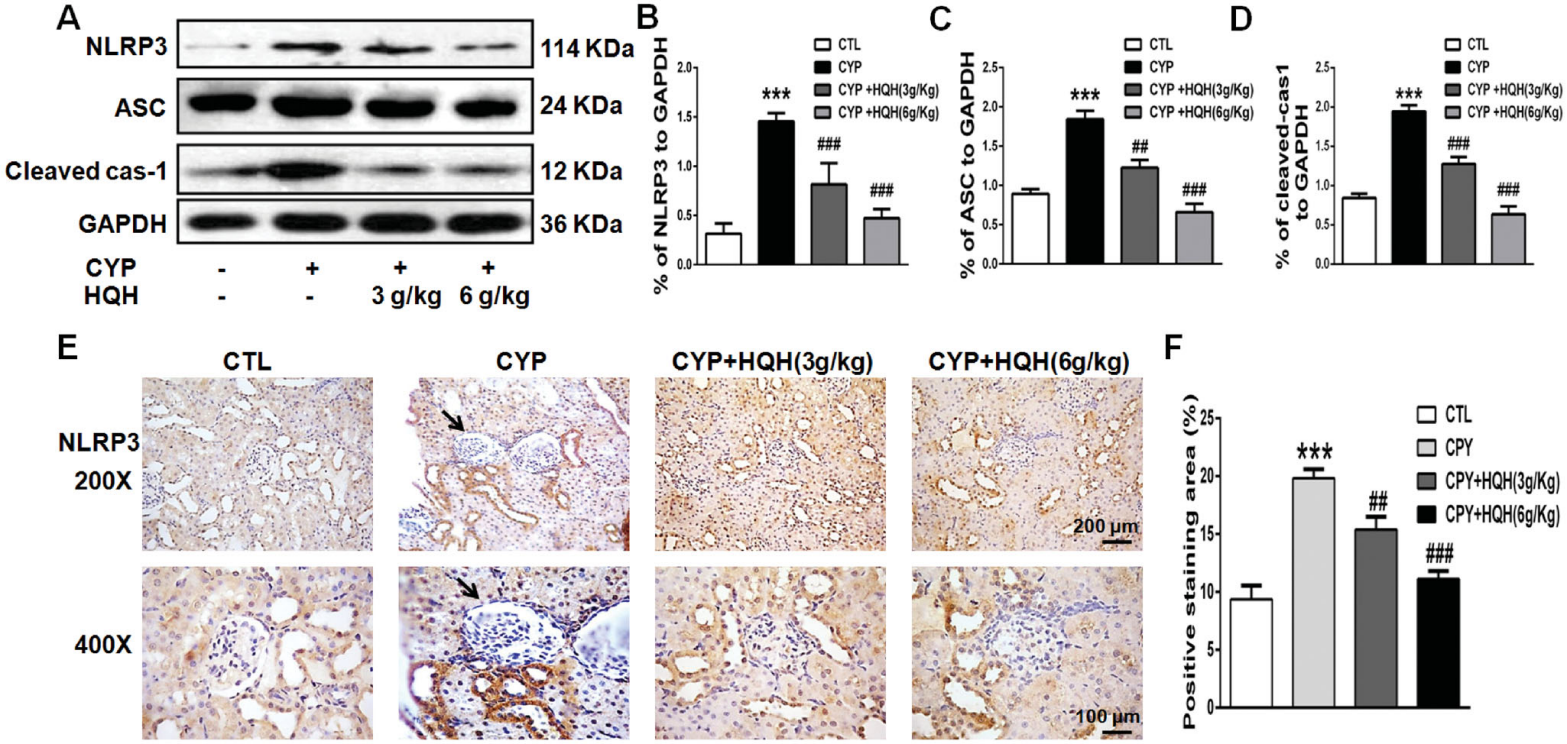
Effect of HQH on protein expression on the NLRP3 inflammatory pathway in the kidneys of CYP-treated rats. (A) The expression levels of NLRP3, ASC and Caspase-1 were determined by western blot representative images of protein bands were shown. (B) The levels of NLRP3. (C) The levels of ASC. (D) The levels of Caspase-1. (E) Immunohistochemistry analysis of NLRP3 and representative images were shown. Data are shown as mean ± SD, *n* = 4. ***p < 0.001 versus the Control group. ##*p* < 0.01 and ###*p* < 0.001 versus the CYP group.

The NLRP3 signalling is closely related to inflammation, the hyper-activation of NLPR3 inflammasome has been shown to mediate a variety of types of kidney damage. However, little is known about the role of NLRP3 in CYP-induced kidney toxicity. To explore the possible mechanism underlying CYP-induced renal changes, proteins in the NLRP3 signalling pathway were detected by western blotting and immunohistochemical staining. CYP prominently activated the NLRP3 signalling pathway (Figure 7(A)), increased the level of NLRP3, ASC, and cleaved-caspase1 by 4.63-, 2.07- and 2.31-fold, while, HQH inhibited the excessive activation (Figure 7(B)).

## Discussion

**Figure 8.**
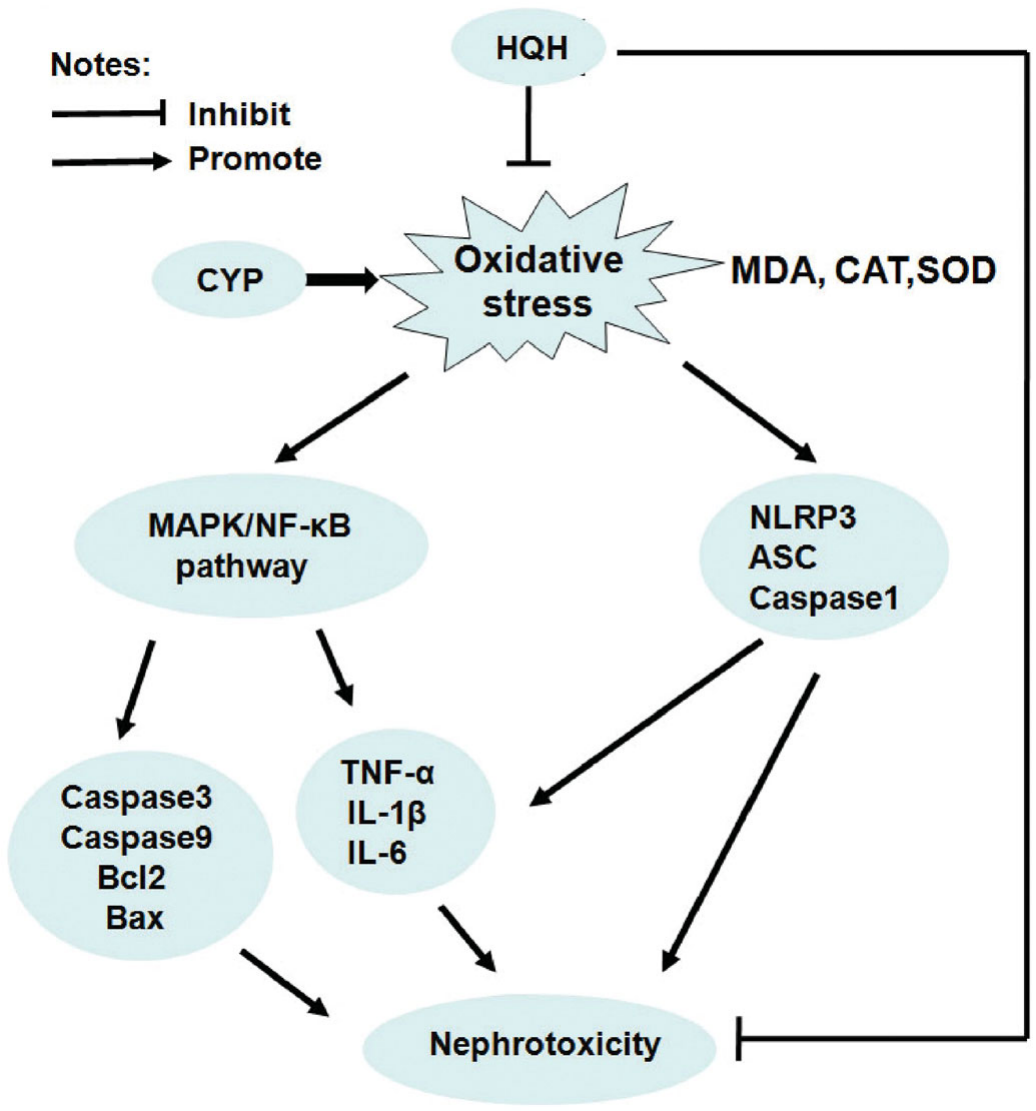
Proposed mechanisms of HQH-mediated protection to CYP-induced nephrotoxicity in rats.

Cyclophosphamide, a widely used anti-cancer drug and immunosuppressive agent, has been shown to cause nephrotoxicity. HQH is an effective Chinese herbal medicine with extensive clinical applications, the most important thing of which is reducing the toxicity of other treatments. This study identified the renoprotective properties of HQH against CYP-induced acute kidney injury and the underlying mechanisms, including its antioxidant and anti-inflammatory activities, as well as its effects on NF-κB/MAPK signalling and NLRP3 inflammasome (Figure 8). To the best of our knowledge, this study is the first to attempt to survey the protective effects of HQH against CYP-induced nephrotoxicity.

Urinary protein contents and serum levels of BUN and CRE are biomarkers of kidney damage, reflecting the functional status of the kidneys. Based on previous report (Liu et al. 2020), we conducted a tissue pathological analysis and assessed the urinary protein contents and serum levels of BUN and CRE to determine whether HQH has a protective role in CYP-induced kidney injury in the current study. The urinary protein contents and serum levels of BUN and CRE increased in rat after CYP treatment, accompanied by morphological changes in kidney tissue sections, including remarkable pyknosis, protein casts, and dilated tubules. However, HQH pre-treatment normalized urinary protein, BUN, and CRE levels and substantially ameliorated the histopathological changes.

Oxidative stress plays a vital role in kidney injury, and intracellular CYP accumulation inhibits cellular antioxidant defense mechanisms (Gunes et al. 2018; Sherif 2018; Mohamed et al. 2020). MDA indicates the level of lipid peroxidation, and SOD, CAT are the most common antioxidants that can eliminate MDA and prevent cell damage. Various antioxidants such as garlic extract, *Olea europaea* L. (Oleaceae) leaf extract, and *Eucalyptus globulus* Labill. (Myrtaceae) polyphenolics protect against CYP-induced nephrotoxicity (El-Sebaey et al. 2019; Ghareeb et al. 2019; Has et al. 2019). Consistently, in our study, HQH effectively reduced the MDA level and improved antioxidant enzyme activities in the kidneys, indicating that HQH defends against CYP-induced oxidative stress in the kidney.

A great deal of evidence has shown that the MAPK and NF-κB pathways are activated by oxidative stress and involved in apoptosis and inflammation during CYP-induced organ toxicity (Nafees et al. 2015; Abdel-Latif et al. 2020; Mohamed et al. 2020; Iqubal et al. 2020a). CYP is also known to increase the serum level of pro-inflammatory cytokines (Kim et al. 2015; Waz et al. 2021). In our study, the mechanisms underlying the effects of HQH on apoptosis and inflammation were explored by examining apoptosis-related protein, the inflammatory cytokines and the inflammatory marker protein NF-κB. HQH is known to reduce apoptosis and promote the survival of kidney cell via the NF-*κ*B signalling pathway (Guo et al. 2018). We found that CYP decreased anti-apoptotic protein Bcl-2 and increased the pro-apoptotic proteins caspase-3 and Bax, as well as significantly upregulated NF-κB/p65 phosphorylation, TNF-α and IL-6, which concurs with previous reports. HQH reduced the CYP-induced apoptotic and inflammatory changes in rats. Additionally, HQH alleviated the phosphorylation of the three subfamilies of MAPK, p38 MAPK, JNK and ERK in CYP-induced nephrotoxicity. This finding preliminarily indicates that one of the protective pathways of HQH in CYP-induced kidney injury is the inhibition of p38 MAPK/JNK/ERK phosphorylation.

Moreover, studies have suggested that NLRP3 can also mediate the inflammatory response and is strongly associated with kidney disease. Previous study showed that CYP leads to the activation of NLRP3 (Lin et al. 2020; Iqubal et al. 2020b). Similarly, in the current study, we found that CYP could significantly increase the expression levels of NLRP3, ASC, and caspase-1 in the NLRP3 inflammatory pathway in the kidney tissues of CYP-treated rats, which were obviously suppressed by the administration of HQH. Therefore, our results show that the anti-inflammatory effect of HQH against nephrotoxicity is partly achieved by inhibiting the NLRP3 inflammasome pathway.

There are two limitations to this study. Firstly, HQH is a Chinese herbal compound, in the present research, we focussed on the protective effect of HQH on CYP-induced nephrotoxicity, whereas, the function of its components is not clear, which should be carefully assessed in the near future. Secondly, we explored the role of oxidative, apoptosis, inflammation, however, investigations on upstream regulatory mechanism are absent, hence, further studies need to be conducted to elucidate the underlying mechanisms.

## Conclusions

Taking the results together, HQH exhibited antioxidative, anti-apoptotic, and anti-inflammatory properties in the kidneys of CYP-treated rats via regulating the MAPK/NF-κB signalling pathways and the level of apoptosis related protein and inhibiting NLRP3 inflammasome activation. These results suggest that HQH is a potent renoprotective agent for preventing the renal toxicity caused by cyclophosphamide and it might be a potential adjuvant for CYP-induced nephrotoxicity.
